# An overview of the role of Niemann-pick C1 (NPC1) in viral infections and inhibition of viral infections through NPC1 inhibitor

**DOI:** 10.1186/s12964-023-01376-x

**Published:** 2023-12-14

**Authors:** Irfan Ahmad, Seyede Narges Fatemi, Mohammad Ghaheri, Ali Rezvani, Dorsa Azizi Khezri, Mohammad Natami, Saman Yasamineh, Omid Gholizadeh, Zahra Bahmanyar

**Affiliations:** 1 Department of Clinical Laboratory Sciences, College of Applied Medical Sciences, King Khalid University, Abha, Saudi Arabia; 2 Faculty of Veterinary, University of Tehran, Tehran, Iran; 3 Student Research Committee, Alborz University of Medical Sciences, Karaj, Iran; 4 Anesthesiology Department, Case Western Reserve University, Cleveland, USA; 5 Faculty of Pharmacy, Tehran University of Medical Sciences, Tehran, Iran; 6 Department of Urology, Shahid Mohammadi Hospital, Hormozgan University of Medical Sciences, Bandar Abbas, Iran; 7 Azad Researchers, Vitro-Biotech, Tehran, Iran; 8 School of Pharmacy, Shiraz University of Medical Sciences, Shiraz, Iran

**Keywords:** Niemann-pick C1, Viral infection, Ebola virus, SARS-CoV-2, NPC1 inhibitor

## Abstract

Viruses communicate with their hosts through interactions with proteins, lipids, and carbohydrate moieties on the plasma membrane (PM), often resulting in viral absorption via receptor-mediated endocytosis. Many viruses cannot multiply unless the host’s cholesterol level remains steady. The large endo/lysosomal membrane protein (MP) Niemann-Pick C1 (NPC1), which is involved in cellular cholesterol transport, is a crucial intracellular receptor for viral infection. NPC1 is a ubiquitous housekeeping protein essential for the controlled cholesterol efflux from lysosomes. Its human absence results in Niemann-Pick type C disease, a deadly lysosomal storage disorder. NPC1 is a crucial viral receptor and an essential host component for filovirus entrance, infection, and pathogenesis. For filovirus entrance, NPC1’s cellular function is unnecessary. Furthermore, blocking NPC1 limits the entry and replication of the African swine fever virus by disrupting cholesterol homeostasis. Cell entrance of quasi-enveloped variants of hepatitis A virus and hepatitis E virus has also been linked to NPC1. By controlling cholesterol levels, NPC1 is also necessary for the effective release of reovirus cores into the cytoplasm. Drugs that limit NPC1’s activity are effective against several viruses, including SARS-CoV and Type I Feline Coronavirus (F-CoV). These findings reveal NPC1 as a potential therapeutic target for treating viral illnesses and demonstrate its significance for several viral infections. This article provides a synopsis of NPC1’s function in viral infections and a review of NPC1 inhibitors that may be used to counteract viral infections.

Video Abstract

**Graphical Abstract:**

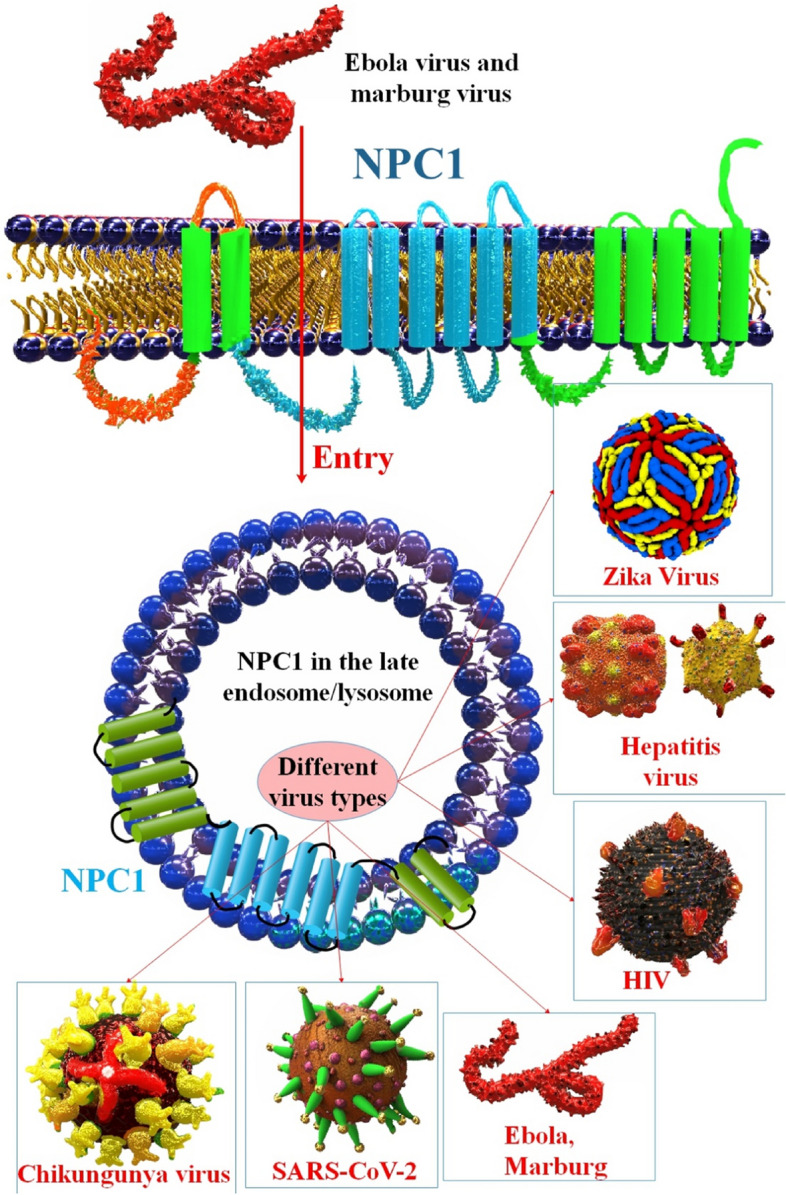

**Supplementary Information:**

The online version contains supplementary material available at 10.1186/s12964-023-01376-x.

## Introduction

The cellular physiological and pathological processes are profoundly impacted by lipids, a large and diverse class of macromolecules [1]. Because of its distinct makeup, the lipid membrane may have either a stiff or flexible shape and a negative or positive curvature, which is essential for membrane rearrangement and the formation of viral replication complexes (RC) [2]. In addition, naturally occurring substances may regulate the infectiousness of some viruses, reducing their infectiousness by altering the viral lipid-dependent attachment to membranes. Enveloped viruses generally infect a host cell by plasma membrane fusion or endocytosis [3, 4]. Although the foundations of how these viruses cause sickness and enter cells are recognized, little is known about how they interact with the lipids of the host cell to acquire their lipid coat. Viral proteins direct assembly and budding outside of the nucleus by binding to the lipid membranes of cells. This talk will explain how newly emerged infections use human cell components to replicate and spread to other cells or patients [5]. In addition, endocytosis is dependent on the presence of lipid raft microdomains, which are distinguished by their unique protein composition. These microdomains act as a docking place and staging area for viruses, allowing them to more easily invade host cells and exfiltrate their DNA. By increasing the local concentration of entry receptors, lipid rafts play a critical role in the viral entry process. Additional viral life cycle steps, including assembly and budding, are affected by these lipid rafts. As a result of viral infections, lipoprotein levels shift [6]. Furthermore, cholesterol is an essential lipid component that is generated by cells in mammals and is dispersed throughout several organs inside the body [7]. Most cholesterol is found inside cellular membranes, which play a critical function as a component that aids in the membrane’s stability, flexibility, and microstructure creation [8]. Cholesterol, a lipid, is essential to reproducing almost all viruses and hence has received a great deal of research. Thus, cholesterol homeostasis modification has recently emerged as a potentially viable technique for combating several kinds of viruses. Further research shows that antiviral methods used to reduce cellular cholesterol impair innate immunity. Therefore, this point should be carefully considered [9]. The function of cholesterol in the propagation of several viruses, including hepatitis, filoviruses, coronavirus, pseudorabies, human immunodeficiency virus (HIV), influenza, and chikungunya virus, has been established [10–16].

Niemann-Pick disease type C (NPC) is characterized by genetic anomalies in the NPC1 and NPC2 genes [17]. These genes are essential in allowing the intracellular movement of cholesterol derived from low-density lipoproteins (LDL) within the endosomes and lysosomes [17]. The NPC1 plays a crucial role in maintaining cellular cholesterol homeostasis by promoting the export of cholesterol from endolysosomes [18]. Recent research has uncovered NPC1’s function within the infection process of several viruses. The majority of the cholesterol in bodies comes through the absorption of low-density lipoproteins. Cholesterol is extracted from these lipoproteins during their processing in late endosomes and lysosomes (LE/Lys) [4]. A cell’s susceptibility to infection by filoviruses like the Ebola virus (EBOV) and Marburg depends, in large part, on NPC1 [19, 20]. Furthermore, the endocytic machinery is used by several viruses to enter cells; these include SARS-CoV-1, MERS-CoV, EBOV, and SARS-CoV-2. Limiting the spread of viruses in bats and increasing human susceptibility to infections have both been linked to genetic changes in the NPC1 gene, which encodes the endo-lysosomal NPC1. Drugs that block NPC1 are effective against various viruses, including SARS-CoV-1 and Type I Feline Coronavirus Infection (F-CoV). Antiviral drug development may benefit from knowing that the NPC1 receptor is a common host target for several viruses. These findings emphasize the importance of NPC1 in a broad range of viral infections and suggest a fresh therapeutic target in the fight against newly developing viruses, which represent a considerable threat to public health and are now a global concern. These pathogens include SARS-CoV-2, EBOV, and the avian influenza virus [21].

The comprehension of the exact mechanisms by which viruses interact with the cells of their hosts is of utmost importance to fully grasp viral pathogenesis and to facilitate developing efficacious vaccines and treatments [22]. For example, the NPC1 receptor acts as an intracellular receptor to allow the entry of the EBOV into the host cells, and it is also thought to be important for the infection of highly pathogenic viruses in which the integrity of cholesterol transport is vital. When the EBOV glycoprotein (GP) binds with NPC1 at the endosomal membrane, the viral material is released into the host cell, starting the infective cycle. Disrupting the NPC1/EBOV-GP interaction may be an attractive strategy for the development of drugs intended to stop viral entry and the ensuing infection. Some of the EBOV inhibitors now available on the market have been proposed to potentially block this interaction [23, 24]. For example, U18666A is a cationic amphiphile that promotes intracellular cholesterol buildup and inhibits NPC1 function. It has shown promising results against a variety of viruses, including Dengue, EBOV, Hepatitis C, SARS-CoV-1, and F-CoV [11, 21, 25, 26]. Nevertheless, due to its toxicity nature, U18666A should only be used in experimental settings [27]. Moreover, it has been shown that some lysosomotropic cationic amphiphiles, including a subset that has received approval from the Food and Drug Administration (FDA), inhibit the entry of EBOV in a way that relies on NPC1. Further investigation is required to explore the possible antiviral properties of these treatments against the same viruses susceptible to U18666A, as well as other viruses such as SARS-CoV-2. Special precautions should be taken while administering FDA-approved drugs such as terconazole and clomiphene, as well as other benzylpiperazine derivatives of adamantane diamide. Imipramine, an antidepressant approved by the FDA, has a similar profile to NPC and demonstrates inhibitory effects on the West Nile, Dengue, Chikungunya, and Zika viruses [21]. In this study, we investigate the characteristics and functions of the NPC1 receptor in its capacity as a cholesterol receptor during viral infections. Furthermore, an examination will be conducted on the many pharmaceuticals and other compounds that inhibit NPC1 and are used in the management of viral infections.

## Cholesterol function in viral infection

Because it modifies the properties of cell membranes and the functions of the proteins present in membranes, cholesterol is an essential component of cellular activities. In addition, cholesterol is transported to other membranes, including the plasma membrane (PM) and the endoplasmic reticulum (ER), after it has been digested in the endo-lysosomal compartment [28]. Additionally, data shows that inflammation may change the number of circulating lipids because membrane cholesterol is a crucial component for facilitating viral entrance into host cells [29]. Recent years have seen an uptick in research highlighting cholesterol’s role in viral infection success. Cholesterol is present in the plasma or endosomal membrane and the viral particle envelope. In reality, the host cell’s cholesterol concentration changes as a result of viral infection, altering the activity of certain mevalonate pathway enzymes and promoting the creation of particular regions within the host cell known as “viral replication organelles.” These organelles exhibit significant quantities of lipids and cholesterol and serve as a substrate specifically for viral propagation [30]. Cholesterol is a signaling molecule that regulates its synthesis, the cell cycle, and the capacity to modify proteins. Some naturally occurring substances may reduce a virus’ infectiousness by messing with the lipid composition of the membrane, which then alters the viral lipid-dependent attachment. To enter the host cell, enveloped viruses often utilize endocytosis or PM fusion. During the endocytosis-mediated process, viruses employ a lipid raft microdomain as a docking point and platform to enter the host cell and release their genome. Lipid rafts influence subsequent viral life cycle stages, including assembly and budding, by raising the local concentration of entrance receptors. Lipoprotein concentrations are altered by viral infections [6].

Many RNA viruses suppress the immune response by using non-structural viral proteins to modify ER membranes to generate partially isolated compartments known as RC, where new viral particles are replicated and synthesized. It’s interesting to note that fatty acids, cholesterol, glycerophospholipids, phospholipids, and sphingolipids are necessary for the creation of the RC (mainly ceramides). For instance, in hepatitis C virus (HCV) infected hepatocytes, the non-structural protein 3 (NS3) is responsible for processing the non-structural section of the viral precursor polyprotein. In addition, the viral infection enhances cholesterol absorption and cholesterol production, and the NS3 protein sequesters the fatty acids synthase to the RC [31]. Many of the deadliest illnesses, including EBOV, influenza virus, *mycobacterium tuberculosis*, cholera, and dengue virus (DENV), employ the endo-lysosomal trafficking mechanism of host cells to enter the cytosol and proliferate. The cytotoxicity of these pathogens may be effectively lowered by abnormalities in endo-lysosomal maturation, trafficking, fusion, or pH homeostasis [32]. Although late endosomes and lysosomes (LE/LYSs) are essential for nutrient absorption and processing, they are also increasingly recognized as sensors of cellular nutritional status [33, 34]. LDLs are primarily responsible for carrying an essential cellular lipid called cholesterol into cells. This lipid is subsequently processed in LE/LYSs to create cholesterol for different cellular sites. Two proteins, NPC1 and NPC2, are necessary to export cholesterol from LE/LYSs. The tiny soluble sterol-binding protein NPC2 is thought to transport cholesterol from LE/LYSs to NPC1 for export, following a well-established paradigm. Importantly, NPC illness results from a deficiency in either NPC1 or NPC2, and it is characterized by an accumulation of cholesterol and sphingolipids in the LE/LYSs of the liver, spleen, and neuronal cells [35].

## Characteristics of Niemann–pick C1 (NPC1)

Keeping cholesterol levels steady in the cell requires a delicate balance between uptake, production, and efflux from various compartments because of its importance to membrane homeostasis and proper cellular function. Cholesterol is transported throughout the cell by the coordinated actions of NPC1 and NPC2 transporter proteins after its absorption and subsequent hydrolysis in the late endosomal organelles. The NPC was initially recognized by Albert Niemann in 1914, and its anatomical description was given by Ludwig Pick; the NPC1 receptor was named after them [20, 36–38]. Mutations in either the NPC1 or NPC2 genes cause NPC. The NPC1 gene encodes a protein that shuttles free cholesterol from the lysosomal interior to the cytoplasm. This protein, NPC1, is localized in the lysosomal membrane. The protein encoded by NPC2 is called NPC2, and it binds free cholesterol in the lysosomal lumen and then offers it to NPC1 [39]. Neurodegeneration, neuroinflammation, and dysmyelination are some of the most severe consequences of NPC1 mutation in the central nervous system (CNS) [40–42]. NPC is an infrequent hereditary condition characterized by a cellular transport deficiency of cholesterol and other lipids. Consequently, there is an atypical buildup of these chemicals in diverse bodily tissues, including brain tissue [43]. Cholesterol buildup in the endo-lysosomal compartment of cells is a biochemical characteristic of NPC. Neurons are the most susceptible to the effects of NPC1 loss, indicating a unique role for this protein in neurons even though endo-lysosomal lipid storage is a problem for all cell types. A separate synaptic function for this protein not predominantly engaged in the degradative process is further supported by the discovery that NPC1 is found not just in neuronal cell bodies, where late endosomes and lysosomes typically dwell, but also in distal axons and synaptosomes. Although NPC1 is present everywhere, its absence has a profound effect on neurons. Changes in cognition and mood are common results of NPC1 mutations in people with NPC [44].

**Fig. 1:**
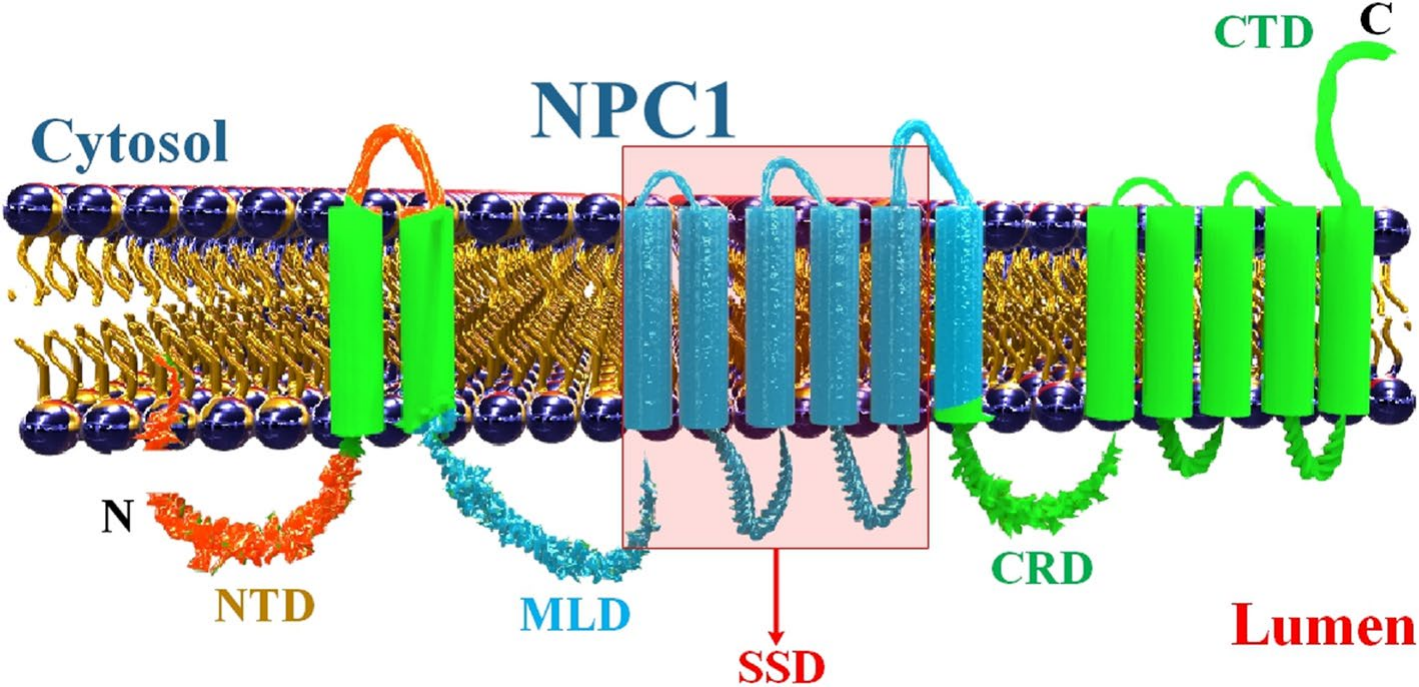
Graphic illustration of the NPC1 protein. Secondary construction diagram of NPC1 in association with the LE/Lys membrane, showing 13 transmembrane domains. NTD, N-terminal domain; MLD, middle luminal domain; SSD, sterol-sensing domain; CRL, Cysteine-rich loop; CTD, C-terminal domain

The intracellular buildup of unesterified cholesterol in lysosomes is the primary consequence of this disorder, which primarily disrupts cholesterol transport. Although NPC sickness is caused by mutations in the NPC1 gene, the NPC2 gene also plays a role in the illness and the cholesterol transport inside cells. The 165 kDa GP with 1278 amino acids mainly found in LE/Lys is encoded by the NPC1 gene [24, 45]. A multi-transmembrane (TM) protein called NPC1 regulates cellular lipid homeostasis by promoting the transport of cholesterol out of lysosomes and late endosomes. Numerous bodily tissues with NPC1 mutations accumulate cholesterol and other lipids. Lysosomal lipid accumulation results in progressive neurodegeneration and neurological impairments in Niemann-Pick type C disease caused by mutations in NPC1 [46]. Cholesterol transport is provided in part by NPC2, a soluble intralysosomal protein that works in tandem with NPC1. Interestingly, the functional interaction of LE/Lys lysophosphatidic acid (LBPA) with NPC2 is essential for cholesterol trafficking at this stage [24, 45]. NPC1 exports cholesterol from LDL that has been taken up by a receptor [47–49]. Additionally, NPC1 is a membrane GP that transiently localizes to lysosomes and trans-Golgi network (TGN) and spends most of its time in late endosomes [50]. Cholesterol is transported from the interior of the LE/LY, where the soluble NPC2 protein is located, to the N-terminal domain of the NPC1 protein, which faces the luminal side of the compartment’s limiting membrane. In turn, NPC1 protein exports cholesterol from the LE/LY for further distribution to various compartments such as recycling endosomes, the ER, Golgi, mitochondria, the PM, and ultimately for extracellular efflux from the cell [51] (Fig. 1).

Although the NPC1 and two protein families have a nearly similar name, they are structurally distinct. As opposed to NPC1, a TM protein with 13 TM domains and a sterol-sensing domain between its third and seventh TM domains, NPC2 is a soluble protein with a myeloid differentiation 2-related lipid recognition protein (ML) domain. Cholesterol transport and metabolism in the late endosomal/lysosomal compartment of the cell are regulated by the NPC1 and two proteins [52]. Since the NPC1 gene product is an insoluble MP, standard enzyme replacement treatment using the recombinant protein is not an option. This highlights the need to develop gene therapy for NPC1. While research continues on viral-based gene therapy, non-viral vector-based gene therapy methods for the brain should be explored. In a study, By utilizing Trojan horse liposomes to encase the plasmid DNA in 100 nm pegylated liposomes and directing them to specified organs using a monoclonal antibody against the mouse transferrin receptor, researchers developed a plasmid DNA technique for gene therapy for NPC1 [53]. A series of antisense oligonucleotides (ASOs) with locked nucleic acid phosphorothioate (LNA-PS) modifications that target NPC1 mRNA may prevent a filovirus GP-pseudo-typed virus from entering cells. Similar to the EBOV, The endocytic machinery is used by SARS-CoV family viruses to enter host cells [46].

## NPC1 in viral infection

For the reproduction of numerous enveloped viruses, NPC1 is necessary. NPC1 is an intracellular receptor for the Marburg virus and EBOV [54–56]. NPC1 plays a role in the reproduction of enveloped viruses by helping to keep cholesterol levels stable. Cholesterol homeostasis is disrupted by suppressing NPC1, which hinders the exosome-dependent release of HCV and blocks the entrance and replication of DENV, chikungunya virus (CHIKV), and African swine fever virus (ASFV). In addition, NPC1 has been linked to the entrance of the hepatitis A virus (HAV) and hepatitis E virus (HEV) that lack a true envelope into cells. When it comes to non-enveloped viral replication, however, NPC1 has mainly been overlooked [10, 16, 57, 58]. Because SARS-CoV-2 may be absorbed by both clathrin- and non-clathrin-mediated endocytosis, various papers have proposed a theoretical function for NPC1 in SARS-CoV-2 infection via an as-yet-undefined mechanism [21, 59, 60] (Table 1).

**Table 1:** Function NPC1 in various viral infections. And a variety of drugs based on NPC1 inhibition to inhibit viral infections

Viral infection	Function NPC1	Drugs and Inhibitors	Ref
Ebola virus	The intracellular receptor for the EBOV is called NPC1. For the subsequent release of the viral contents to the infected cells, the cleaved GP (GPcl) must first bind to NPC1 via domain C.	U18666A, Imipramine, and Adamantane dipeptide piperazine 3.47, triazole thioether MBX2270, and aminoacetamide sulfonamide MBX2254	[54, 61–63]
Marburg viruses	As a membrane receptor for MARV, NPC1 is required for viral capsid release into the cytoplasm and GP2-dependent fusion of the viral and endosomal membranes.	N-heterocyclic bornyl esters, verapamil, Apilimod	[64–66]
African swine fever virus (ASFV)	There was evidence that these viral proteins interacted with NPC1 and Lamp-1 and − 2, found in the cellular endosome. Additionally, silencing these proteins hampered ASFV infection.	–	[67]
HIV	Similar to the downregulation of ABCA1 with HIV-1 infection, NPC1 protein expression may likewise be downregulated.	U18666A, and Cepharanthine	[68–71]
SARS-CoV-2	protease for SARS-CoV-2 fusion, but its activity is hindered by NPC-related lysosomal membrane permeabilization, which causes cathepsin L leakage, and by the elevated intra-lysosomal pH found in NPC.S-CoV-2.	U18666A, Cepharanthine	[15, 72]
Type I FCoV	NPC1 plays an important role in type I FCoV infection.	U18666A, Cepharanthine, and Posaconazole	[15, 73]
HCV	To attract cholesterol to the viral replication organelle, where it helps MW functioning, HCV hijacks lipid transfer proteins such NPC1 at ER-late endosome/lysosome membrane contact sites.	U18666A, Itraconazole, and Posaconazole	[74, 75]
CHIKV	These cells were transduced with CHIKV-glycoproteins pseudo-typed particles, proving that NPC1 and NPC2 deficiencies impact the entry/fusion phases of the CHIKV life cycle.	U18666A, and Imipramine	[10]
YFV	NPC1 plays an essential role in YFV.	U18666A, and Posaconazole	[31]
DENV	To support cholesterol trafficking and metabolism, both NPC1 and NPC2 proteins dwell and collaborate closely in the late endosomal/lysosomal compartment of cells.	U18666A, Imipramine, Itraconazole, and Posaconazole	[52, 75, 76]
ZIKV	Although we lack direct evidence of emetine’s interaction with NPC1, there is a possibility that NPC1 plays a role in emetine’s inhibitory impact on the viral entrance.	U18666A, Imipramine, and Posaconazole	[77, 78]
Reoviruses	NPC1 regulates the level of cholesterol in endosomes, which is essential for the efficient release of reovirus cores into the cytoplasm.	Hydroxypropyl-β-cyclodextrin	[57]

### Ebola virus

The Ebola virus disease (EVD) is a significant threat to human and nonhuman primate health [79]. The RNA virus genus Ebolavirus, which is a member of the family Filoviridae, is what causes EVD [80]. Transmission of EVD, a severe and highly infectious viral hemorrhagic fever (VHF), occurs by infected body fluids coming into contact with the skin or mucous membranes [81]. The polymerase cofactor and interferon (IFN) antagonist viral protein 35 (VP35) of EBOV is an essential component in viral replication. By obfuscating the non-self-5′-PPP dsRNA, VP35 suppresses retinoic acid-inducible gene I (RIG-I) activation and IFN- generation. It is shown that inhibiting the interaction between VP35 and dsRNA, which inhibits IFN-β production, is a legitimate therapeutic target [82]. The viral GP is located on the surface of EBOV and is responsible for mediating the virus’ entrance into host cells. GP is a class I fusion protein with two halves, GP1 for binding to receptors and GP2 for fusing. Internalization through macropinocytosis and subsequent trafficking to late endosomes/lysosomes follows the attachment of virus particles to the cell surface. The highly glycosylated mucin-like domain and glycan cap of GP1 are removed in this compartment by the low-pH activated endosomal cysteine proteases cathepsin B and cathepsin L, resulting in a cleaved version of GP1 (about 19 kDa). The receptor-binding site for the EBOV endosomal receptor, NPC1, is made visible by the cleavage of GP1. Following its attachment to NPC1, GP2 promotes the fusing of the viral and endosomal membranes by an as-yet-unidentified process that is more intricate than that of the majority of other enveloped viruses that fuse at low pH levels [83]. NPC1 knockout cells are impervious to the virus, and NPC1 knockout animals are resistant to lethal EBOV infection, supporting this crucial necessity for NPC1 protein in EBOV replication [84–86].

**Fig. 2:**
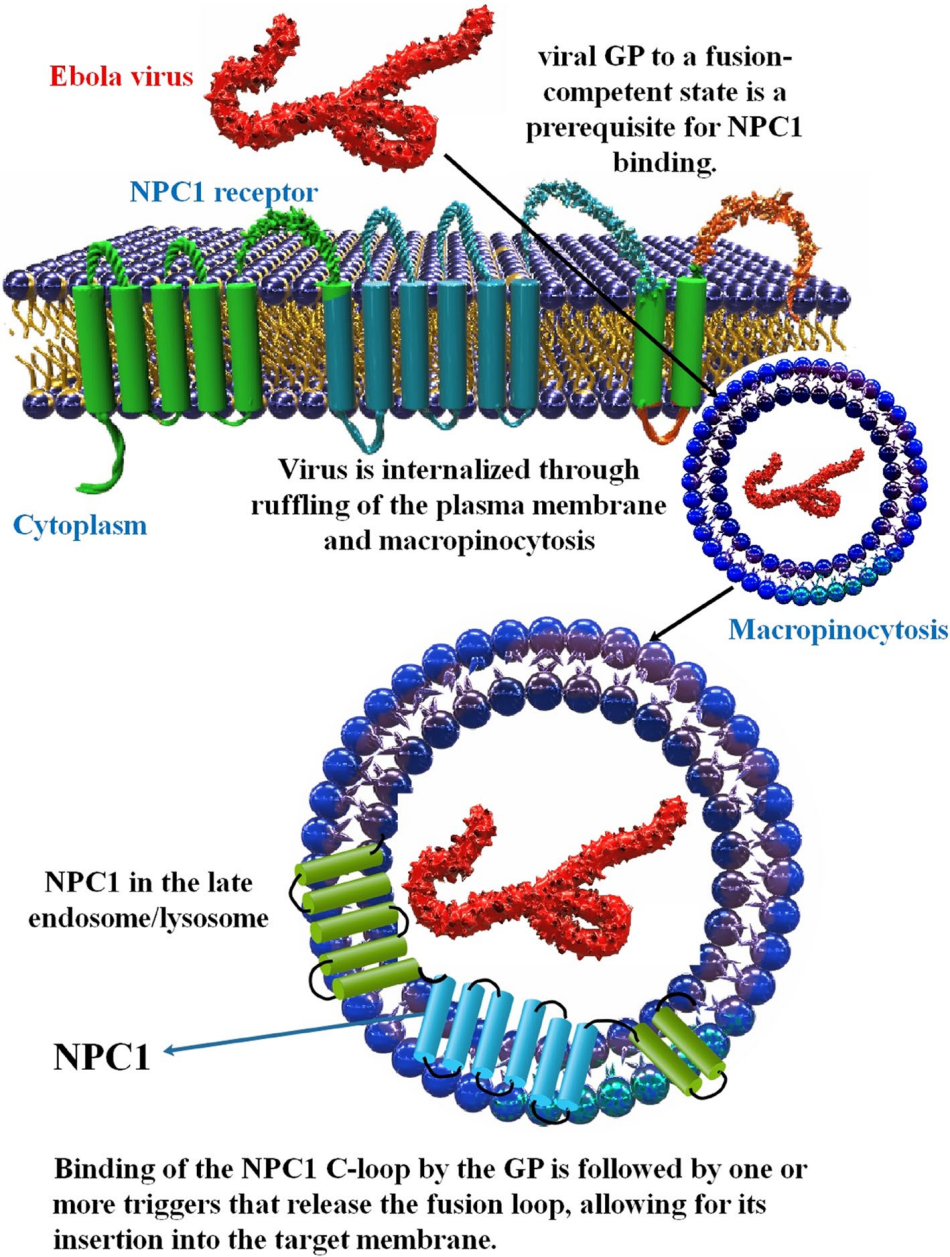
Ebola virus replication by binding to NPC1 receptor. EBOV enters by ruffling the plasma membrane and macropinocytosis. The EBOV receptor binding domain (RBD) interconnects with NPC1 in the LE/Lys

Proteolytic processing of a precursor molecule results in two subunits, GP1 and GP2, which are then incorporated into the envelope of the virus obtained from the host cell [87]. Attachment is typically mediated by GP1, 2. However, phosphatidylserines in the viral envelope interact with the host attachment protein T-cell immunoglobulin and mucin domain 1 (TIM-1), suggesting that filoviruses may use apoptotic mimicry to gain entrance. Micropinocytosis is the primary route by which attached viruses are internalized and transported to endolysosomes [56, 88, 89]. Recent single-molecule Förster resonance energy transfer (FRET) research from the Munro lab reveals that changes in GP2 conformation may occur in response to acidic pH, Ca2+, and NPC1 binding [90]. Further study demonstrated that T-cell immunoglobulin and mucin domain 1 (TIM-1) and NPC1 interacted and colocalized in the intracellular vesicles where EBOV GP-mediated membrane fusion occurs. The TIM-1-specific monoclonal antibody (MAb) M224/1 could block GP-mediated membrane fusion and inhibit TIM-1 binding to NPC1, indicating that this interaction is critical for filovirus membrane fusion. Furthermore, MAb M224/1 effectively blocked the entrance of all known filovirus species into cells [91] (Fig. 2).

### African swine fever virus (ASFV)

The lethal illness caused by ASFV has moved beyond its original African range and has a significant socioeconomic effect in Europe, Asia, and Oceania. Endocytosis is the route through which ASFV enters the cell, and it is in the endosome that the virus acquires its infectious properties. Replication of the virus requires the release of its DNA into the cytoplasm, which can only occur when the internal viral membrane has fused with the endosomal membrane [67, 92–94]. The virus is then taken into the cell using endocytosis and macropinocytosis mediated by clathrin and dynamin. Internalization and incorporation into the endocytic pathway are standard procedures for viruses like ASF virion. Decapsidation exposes the inner viral membrane, fusing with the late endosome’s limiting membrane. Thus, arriving virions must undergo fusion to escape endosomes and enter the cytoplasm. This process is both fascinating and crucial to infection since it allows viral nucleic acid to enter the cytoplasm and replicate. E248R and E199L, two proteins of ASFV, are thought to have a role in fusion since they are found on the outer surface of the viral membrane. Proteins from the cellular endosome like NPC1 and lysosomal membrane (Lamp-1) were revealed to interact with the viral proteins. ASFV infection was also hampered by the silencing of these proteins [67]. Furthermore, E248R’s TM domains connect to the loop C of NPC1 at the same domain as the EBOV binding site. These ASFV proteins are likely essential for membrane fusion due to their interactions with other molecules. Vero cells engineered by Clustered Regularly Interspaced Short Palindromic Repeats (CRISPR) to lack the NPC1 protein, which confers resistance to EBOV, showed considerably less ASFV infection after being exposed to EBOV. NPC1 KO cells had decreased infectivity and replication of ASFV, followed by smaller viral factories that lacked the characteristic cohesive architecture between endosomes and viral proteins. Researchers found that knocking down NPC1 in a cell increased NPC2 levels, compensating for the reduced ASFV infection seen when silencing NPC2 in Vero cells using short hairpin RNA (shRNA). Deletion of the endosomal protein NPC2, which is linked with the virus, also reduced infectiousness [95].

### HIV

Several points in the lifecycle of HIV-1 infection rely on cholesterol pathways for their success. A study employed a novel NPC2-deficient cell line (NPCD55) that, upon HIV infection, accumulated Gag and reduced NPC1 expression. Infected NPCD55 cells treated with the cholesterol efflux-inducing medication TO-9013171 had their viral infectivity lowered to baseline levels [68]. Inhibition of NPC1 has been linked to the accumulation of the HIV-1 viral gag protein in the endo-lysosomal compartment, which in turn significantly reduces virion release. Negative regulatory factor (Nef), an auxiliary protein of HIV-1, was shown to trigger genes involved in cholesterol synthesis and homeostasis, suggesting that NPC1 might be a therapeutic target for treating HIV-1 infections, which rely heavily on host cell cholesterol levels [84]. In HIV-infected macrophages, Nef decreases ATP-binding cassette transporter A1 (ABCA1) function. Cholesterol efflux is inhibited, and intracellular cholesterol accumulates due to ABCA1 inhibition. In addition, microRNA-33 (miR-33) substantially decreases NPC1 protein production and blocks ABCA1-mediated cholesterol efflux. Since HIV-1 infection of NPCD55 cells induces sterol regulatory element-binding protein-1 (SREBP) expression, miR33 may be responsible for the reduced expression of NPC1. Direct interaction between HIV-1 Nef and ABCA1 and downregulation of ABCA1 expression have reduced cholesterol efflux. Downregulation of NPC1 protein expression may occur like that of ABCA1 post-HIV-1 infection [68–70].

### SARS-CoV-2

**Fig. 3:**
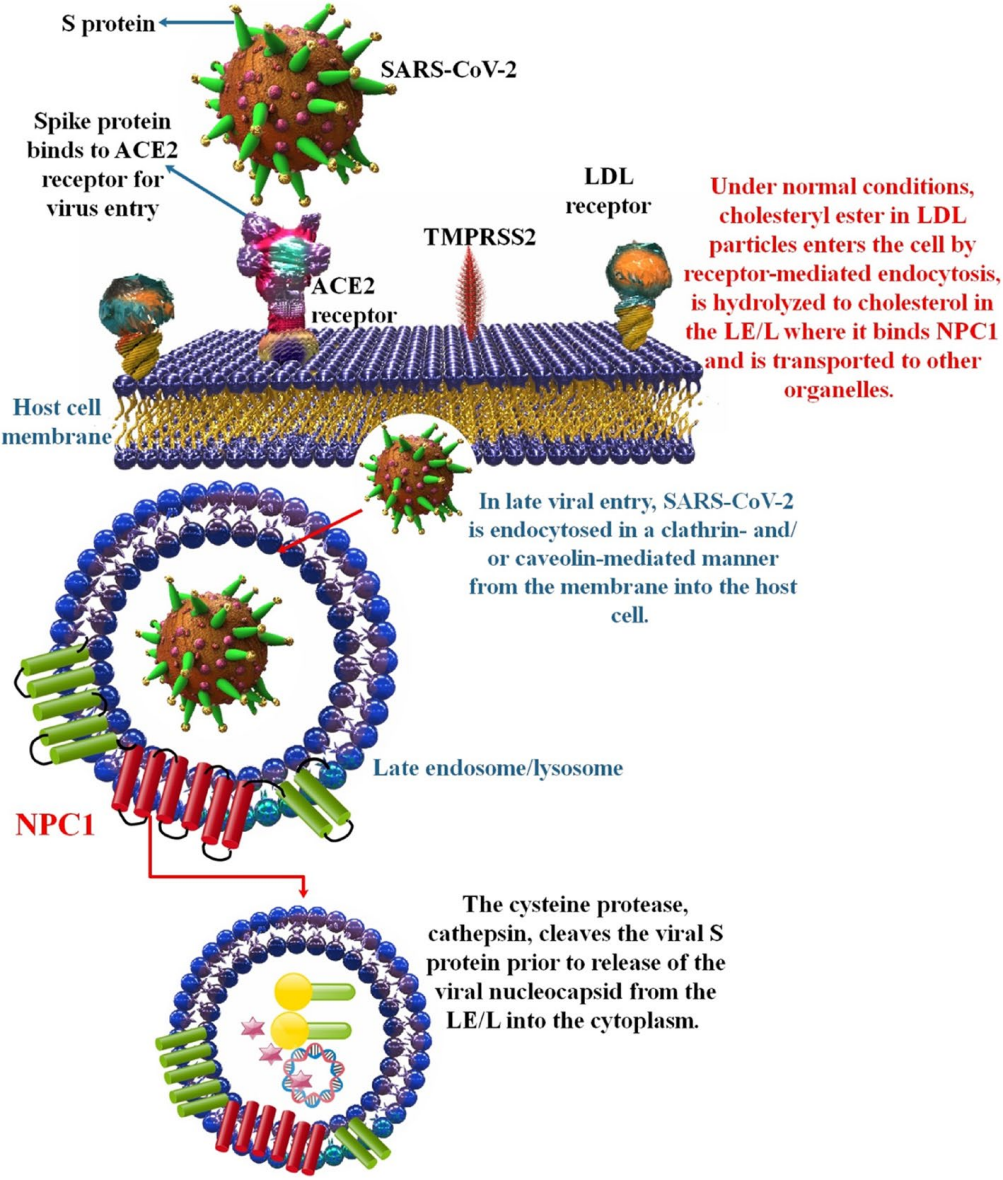
NPC1 function in SARS-CoV-2 entry. By binding to the ACE2 receptor, SARS-CoV-2 can enter the cell during early entry. Instead, for late viral entrance, this virus is endocytosed in a clathrin- and/or caveolin-intermediated method from the membrane into the host cell

The SARS-COV-2 virus, which causes COVID-19 illness, has rapidly spread over the globe. SARS-COV-2 is the biggest known RNA virus, with genomic sizes between 27 and 32 kb. The 3′-end ORFs encode the structural proteins, such as the nucleocapsid (N), membrane (M), envelope (E), and spike (S) proteins. The SARS-CoV-2 virions attach to the host cell through the S protein, which binds to the Angiotensin-converting enzyme 2 (ACE2) [96–99]. In addition, it has been shown that SARS-CoV-2 may enter the cell through PM fusion and/or endosomal entry, depending on the availability of proteases. Since SARS-CoV-2 may be absorbed by both clathrin- and non-clathrin-mediated endocytosis, there has been speculation that NPC1 plays some kind of function in SARS-CoV-2 infection, albeit the exact nature of that involvement remains unclear [60, 72, 100]. Additionally, the elevated disintegrin and metalloprotease 17 (ADAM17) levels in the plasma membrane of NPC cells promote ACE2 shedding, which in turn inhibits viral docking in the plasma membrane of host cells. Cathepsin L is a crucial protease necessary for the effective fusion of SARS-CoV-2. Still, its activity is inhibited by NPC-related lysosomal membrane permeabilization, which results in cathepsin L leakage, and by the elevated intra-lysosomal pH found in NPC [72]. Intriguingly, NPC1 was shown to be a potential therapeutic target for several enveloped viruses, including influenza A virus (IAV) and SARS-CoV-2. Reduced viral replication was observed in NPC1-deficient cells, and it was hypothesized that increased endo-lysosomal cholesterol levels impeded virus uncoating by interfering with the correct insertion of the fusogenic IAV hemagglutinin domains and the S protein. Numerous in vitro studies have used the cell-permeable hydrophobic polyamine U18666A, a small-molecule NPC1 inhibitor, to examine the role of NPC1 in diverse cellular processes. However, therapeutic use is unlikely because of the compound’s high toxicity. As an interesting side note, itraconazole has also been demonstrated to directly bind and inhibit NPC1, suggesting that it might be a promising option for NPC1 targeting tactics through medication repurposing. In support of this idea, cells treated with itraconazole produced much fewer IAV and EBOV offspring, and a positive treatment result was validated in vivo using a mouse model of IAV infection. The antiviral effects may be attributable to itraconazole’s potential to induce IFN-1, a key component of antiviral immunity. Itraconazole showed antiviral potential in a three-dimensional cell culture model for SARS-CoV-2 infection, but in a hamster infection model, it had no impact [84]. Other research has shown a previously unknown relationship between N and NPC1, a protein transporting cholesterol. Further, it was demonstrated that the carbazole SC816 and the sulfides SC198 and SC073, all of which were known to interact with NPC1, were effective in inhibiting SARS-CoV-2 infection in human cell infection models with a high selectivity index. Over 95% suppression of SARS-CoV-2 infection was seen with an IC50 in the low micromolar range for imipramine, sulfides (SC073, SC198), and carbazole SC816, but benzothiazepine SC397 exhibited no significant inhibition [60]. Drugs that target late endosome proteins, such as cathepsin L, two-pore channel 2 (TPC2), and FYVE finger-containing phosphoinositide kinase (PIKfyve), are also effective against SARS-CoV-2 pseudovirions. Antibodies against these proteins are effective in halting infection, suggesting they are pivotal host factors for endocytosed Contamination by SARS-CoV-2 [101, 102] (Fig. 3).

### Hepatitis

#### Hepatitis a virus

HAV is firstly transmitted through fecal-orally following near contact with a sick person. It is the most common reason for viral hepatitis globally, usually leading to severe and self-limited signs; however, fulminant hepatic failure and death can happen [103]. HAV has various properties that make it exclusive among the Picornaviridae, especially in terms of its mechanisms of polyprotein processing and virion morphogenesis, and is presumably involved in its pathobiology [104]. This virus RNA genome contains a single, long, open reading frame (ORF) flanked via a 5′-UTR and 3′-UTR. The HAV interior ribosomal entry site (IRES) is placed in 5′-UTR and translates as HAV proteins in a cap-independent method. ORF encodes structural (Viral Protein (VP4, VP2, VP3, VP1, and pX)) and non-structural (2B, 2C, 3A, 3B, 3C, and 3D) proteins. One big immature protein, encoded via open reading frames (ORF), is firstly cleaved into at minimum ten mature proteins via HAV 3C protease. HAV 3D has an RNA replicase, which is crucial for HAV replication [105]. Increasing evidence suggests that NPC1 and phosphatidylserine receptor-HAV cellular receptor 1 (HAVCR1) have roles in quasi-enveloped HAV infection via a process that facilitates the transfer of genetic material into the cytoplasm but remains unexplained. Because of this, quasi-enveloped HAV and quasi-enveloped HEV (eHEV) use the HAVCR1-NPC1 route to enter cells [106]. Recently, a third cell-to-cell transmission mode of HAV was reported: the spread of capsid-free RNA genomes, a copious payload of quasi-enveloped virions. To be taken up by clathrin-mediated endocytosis, the quasi-enveloped virions, which are released from exosomes, need phosphatidylserine molecules in their membranes and contact with the extracellular domain of the HAVCR1, which was previously identified as an HAV receptor. The intraluminal region of the cholesterol transporter (NPC1) mediates fusion between the exosome and late endosome membranes, which releases the capsid-free RNA payload into the cytoplasm [107]. The phosphatidylserine receptor HAVCR1 and the cholesterol transporter NPC1 are involved in the transportation of cargo from exosomes (exo) of HAV-infected cells (exo-HAV), according to research using clathrin-mediated endocytosis. Using CRISPR-Cas9 knockout technology, researchers demonstrate that the membrane fusion and transfer of RNA from exo-HAV into the cytoplasm need the contact of these two lipid receptors, which occurs in the late endosome. A study implies that an envelope GP is not required for viral infection via this exosome mimicking mechanism since exo-HAV enables HAV infection through the HAVCR1-NPC1 route employed by EBOV to infect cells. Capsid-free viral RNA in the exosome lumen, rather than endosomal uncoating of HAV particles present in the exosomes, is the primary cause of exo-HAV infectivity, as determined by methylene blue inactivation of non-encapsidated RNA. In contrast, HAV particles are infectious regardless of pH and need HAVCR1 or another receptor whose identity we do not yet know, but not NPC1. This work reevaluates the function of envelope glycoproteins in infection by demonstrating that exosomes and viruses employ similar envelope-glycoprotein-independent fusion processes [108].

#### Hepatitis B virus

The infection and life cycle of the hepatitis B virus (HBV) rely on lipid metabolism, particularly cholesterol metabolism, in the host. One method used to combat viral infections is the manipulation of genes and proteins involved in cholesterol metabolism in the host. Research into the use of cholesterol-lowering medications, especially 3-hydroxy-3-methylglutaryl coenzyme A (HMG-CoA) reductase inhibitors, for treating hepatitis virus infections has shown encouraging results [109]. Nonalcoholic steatohepatitis (NASH) has been linked to lipid metabolic dysregulation and inflammation in the liver, but the mechanisms behind these associations are poorly understood. NASH is associated with the HBV x protein (HBx). Researchers showed that hepatocyte-derived prostaglandin E2 (PGE2) expressed by HBx caused an imbalance in macrophage polarization through prostaglandin E2 receptor 4 (EP4) in in vitro, ex vivo, and in vivo models. The M1-type polarization of macrophages, which was caused by ER oxidoreductase-1-like protein-dependent ER stress, was demonstrated to be associated with the hepatic NASH phenotype caused by HBx. To summarize, HBx activated the mammalian target of rapamycin (mTOR), which in turn boosted NPC1/oxysterol-binding protein-related protein 5 (ORP5), which in turn accelerated cholesterol transport from the lysosome to the ER. This provides a valuable resource for evaluating targeted therapeutics for HBx-associated NASH and screening prospective biomarkers in the macrophage mTOR-cholesterol homeostasis-polarization regulation signaling pathway [110].

#### Hepatitis C virus

The HCV life cycle is orchestrated by the coordinated actions of ten viral proteins. p7, non-structural protein 2 (NS2), and the replicase proteins NS3, −4A, −4B, and -5A and -5B assist in building the virion’s significant components, core, E1, and E2. HCV, like other positive-strand RNA viruses, induces extensive membrane rearrangements in infected cells, referred to as the membranous web. As the most numerous membrane structures in HCV-infected cells, double-membrane vesicles (DMVs) form at a rate directly proportional to the rate at which viral RNA is replicated. More evidence that DMVs are where HCV RNA replication occurs comes from the discovery that affinity-purified DMVs contain an active viral RNA replicase [111–113]. Cholesterol entrapment in lysosomal vesicles and a related decrease in cholesterol abundance at sites containing the viral replicase component NS5A were determined to be the outcome of knockdown or pharmacological inhibition of NPC1, according to a study. Unesterified cholesterol accumulated in the perinuclear area of untreated HCV-infected cells, partly colocalizing with NS5A at DMVs, suggesting that cholesterol was transported to the viral replication organelle through NPC1-mediated endosomal transport. Cholesterol is thought to be an essential structural component of DMVs, and research has shown that reduced NPC1-dependent endosomal cholesterol trafficking compromises MW integrity. HCV seems to hijack lipid transfer proteins like NPC1 at the ER-late endosome/lysosome membrane contact sites to attract cholesterol to the viral replication organelle, which assists with MW activity [74]. Cationic amphiphile treatment, like NPC1 knockdown, drastically inhibited the replication of stable subgenomic replicons of genotype 1b while having little effect on the RNA replication of the replicon of genotype 2a. This led researchers to hypothesize that NPC1, maybe in collaboration with StAR-related lipid transfer domain-3 (STARD3) and Oxysterol-binding protein-related protein 1 (OSBPL1B), is involved in the transport of cholesterol from endosomes to cytosolic sterol acceptors. Consistent with this, researchers discovered that the diameters of DMVs were smaller, and their shapes were more erratic after being treated with cationic amphiphiles, which impair endosomal cholesterol transport. Their data imply that cholesterol trafficking via the endosomal route is critical for maintaining membranous web integrity in DMVs. Still, it is possible that reduced cholesterol levels make DMV membranes more brittle and, hence, more prone to EM preparation errors [74].

#### Hepatitis E virus

Around the globe, acute hepatitis is often brought on by HEV infection. HEV has been regarded as non-enveloped since its discovery in the 1980s. While virions found in bile and feces do exist in this form, it is now known that virions circulating in the circulation also exist in a membrane-associated or “quasi-enveloped” form (eHEV). eHEV particles are infectious, but since they lack the conventional envelope proteins, they uniquely infect cells [106, 114, 115]. Researchers began investigating the virus’s entry mechanism since eHEV virions lacked viral proteins on their surface. Researchers discovered that the effectiveness of eHEV attachment to the cell was significantly inferior to that of non-enveloped HEV virions, necessitating a longer inoculation duration to reach its maximal infectivity. An examination of cellular internalization mechanisms revealed that clathrin-mediated endocytosis is the main route by which eHEV enters cells. Contrary to non-enveloped HEV virions, eHEV entry requires the small GTPases Rab5 and Rab7, and inhibiting endosomal acidification made eHEV infectivity ineffectual. Low pH, on the other hand, did not effectively remove the coating from the eHEV on its own, suggesting that further entry techniques are required. eHEV infectivity was dramatically reduced in cells treated with a lysosomal acid lipase inhibitor or in cells devoid of NPC1 lends credence to this theory. These results indicate a potential novel viral entry mechanism in which the quasi-envelope is destroyed within the lysosome before the virus uncoils [116]. The breakdown of lipid membranes in lysosomes is a complicated process. The extraction of cholesterol from lipids by NPC1 is an essential step in breaking lipid membranes. A study showed that without substantially impacting HEV infectivity, NPC1 depletion lowered eHEV infection by 50%. Furthermore, eHEV infectivity was reduced in a dose-dependent manner in cells pretreated with a specific inhibitor of lysosomal acid lipase (LAL), an enzyme crucial for lipid metabolism because it hydrolyzes cholesteryl esters and triglycerides in lysosomes. Still, non-enveloped HEV infectivity was unaffected [114, 117]. Depletion of the lysosomal cholesterol transporter NPC1 decreased eHEV infectivity, providing further evidence that NPC1 is involved in the extraction of cholesterol from the eHEV membrane during eHEV entrance. Since the loss of NPC1 did not affect HEV entry, it is doubtful that NPC1 is a receptor for eHEV, as it was for ebolavirus. Inhibition of the lysosomal acid lipase also reduced eHEV infectivity, suggesting that the eHEV membrane is degraded in lysosomes. Patients with lysosomal storage disorders, where mutations in NPC1 and LAL are expected, may have an innate resistance to HEV infection [61, 116, 118, 119].

### Chikungunya virus

The symptoms of CHIKV infection, spread by mosquitoes of the genus *Aedes* spp., include myalgia, joint pain, a rash, and acute asthenia sickness [120, 121]. In the *Togaviridae* family, CHIKV is classified as an alphavirus due to its icosahedral-shaped virions (about 70 nm in diameter) [122]. The latter open reading frame (ORF) is responsible for translating six structural proteins (capsid (C) protein, E1, E2, and E3 glycoproteins, and 6 K/TF proteins) that function together to facilitate viral particle formation, attachment to, and entrance into, their respective target cells [123, 124]. These cells, which have mutations in the NPC1 or 2 proteins, were infected with CHIKV and showed dramatically decreased viral replication and titers. In addition, transduction of these cells with CHIKV-GPs pseudotyped particles showed that both NPC1 and 2 deficiencies impact the entry/fusion phases in the CHIKV life cycle [125]. The same seems true for the Vesicular Stomatitis Virus. However, NPC1 deficiency did not affect transduction mediated by Gibon Ape Leukemia virus-pseudo-typed particles that fuse directly with the PM [126]. These results prove that NPC1 and 2 work together inside LE/Ls23 to traffic cholesterol. It is unknown how dysfunctional NPC1 or 2 affects viral fusion at this time. Furthermore, pretreatment with the class II cationic amphiphilic chemical U18666A or therapy with the FDA-approved antidepressant medicine imipramine resulted in nearly 100% suppression of viral replication and generation at the highest dosage utilized with no harmful effects [4]. The fusion and replication phases of the viral life cycle were discovered to be influenced by imipramine. Using fibroblasts from NPC patients where CHIKV replication is inhibited provided additional confirmation that cholesterol availability plays a crucial role in the CHIKV life cycle. Zika, West Nile, and DENV, as well as others in the *Flaviviridae* family, were all severely suppressed by imipramine [10].

### Flaviviruses

The *Flavivirus* genus is part of the more prominent *Flaviviridae* family, including the *Hepacivirus* and *Pestivirus* genera. Viruses, including yellow fever virus (YFV), Japanese encephalitis (JEV), DENV, West Nile, and tick-borne encephalitis (TBE), are all transmitted to humans by arthropods. *Flaviviruses* are a family of small enveloped RNA viruses. A flavivirus consists of a nucleocapsid that contains genomic RNA and numerous copies of the C protein, surrounded by an envelope that includes 180 copies of the E and M proteins [127–129]. Cholesterol has been shown to have a crucial role in both mammalian and insect vector models of flavivirus infection, it was revealed recently. Viral entrance, replicative complex formation, assembly, egress, and regulation of the IFN-I response are all aided by the host’s cholesterol levels during infection with DENV, ZIKV, YFV, and WNV. Cholesterol production and absorption are affected, as are cholesterol receptors, as a result of crucial alterations in cellular metabolic processes. Because flavivirus reproduction requires increased cholesterol uptake and synthesis, a combination of medications that limit uptake and synthesis may result in a more effective healthcare distribution alliance (HDA) treatment for flavivirus inhibition. Moreover, it has been shown that the chemical substance U18666A may block NPC1 activity, hence reducing infection with numerous flaviviruses, including ZIKV, WNV, YFV, and DENV [31].

Five members of the ML and NPC1 families of lipid-binding proteins have shown an increase in transcript abundance in transcriptome analysis studies in response to DENV infection. These lipid-binding proteins are likely implicated in mosquito-virus interactions since DENV is an encapsulated virus with a lipid-based outer coat [52] (Table 1).

#### Zika virus (ZIKV)

Once thought to pose minimal harm to human health, the Zika virus became a primary concern during the 2015–2016 epidemic [130]. Although some cases of Zika virus infection are mild or asymptomatic, others may result in serious health issues such as Guillain-Barré syndrome in adults and microcephaly in babies exposed to the virus during pregnancy. Direct infection of neural progenitor cells (NPCs) seems to be the primary source of ZIKV-induced microcephaly; however, other cell types may also play a role. ZIKV infection causes cell cycle dysregulation in NPCs, suppressing cell proliferation and early neuronal differentiation [131]. Since lipids are crucial to flavivirus infection, the dose-dependent lipid buildup seen when emetine is administered raises the possibility of yet another mechanism by which the drug interferes with ZIKV infection. The action of emetine may be partly due to a decrease in autophagic flux and an inhibition of autophagy, both of which may result from lysosomal dysfunction. Researchers do not have direct evidence of an interaction between emetine and NPC1, but it is fair to hypothesize that NPC1 plays a role in emetine’s inhibitory impact on the viral entrance. To further understand how ZIKV infection affects lysosomal protein activity, more research is required [77].

### Reoviruses

The dsRNA genome of the enteric, non-enveloped reovirus is segmented. Typically, reovirus infections begin in infancy and continue throughout a person’s life. Most people with reovirus infections don’t have any symptoms at all [132]. The reovirus virion comprises two protein layers: an exterior capsid and an inner core. Reovirus cell entrance is facilitated by the outer-capsid proteins μ1, σ1, and σ3, as well as the core protein λ2. During cell entrance, viruses undergo a process of increasing proteolytic disassembly, producing infectious subversion particles (ISVPs) and cores that are distinct from intact viruses in terms of their protein structure and content [133]. Using genome-wide CRISPR/Cas9 and short interfering RNA (siRNA)-based cell-survival screens, researchers found that NPC1 was a necessary host component for reovirus infection. Early phases in reovirus infection have been extensively studied, including receptor binding, acid-dependent proteolytic disassembly, and ISVP-to-ISVP* conversion. To investigate NPC1’s role in reovirus infection, scientists employed CRISPR/Cas9 to knock down the gene for NPC1 expression in human brain microvascular endothelial cells (hBMECs). NPC1 was shown to be unnecessary for viral binding to cell-surface receptors, viral particle internalization, and viral outer capsid disintegration. Reovirus cores can’t enter the cytoplasm without NPC1, however. Cholesterol buildup in endosomes is decreased by 2-hydroxypropyl-β-cyclodextrin (HβCD) treatment, and reovirus infectivity is restored in NPC1 KO cells. Researchers demonstrated that NPC1 mediates endosomal cholesterol homeostasis, which is necessary for the effective release of reovirus cores from endosomes into the cytoplasm [57].

## Reuse of NPC1 inhibitors for antiviral therapy

It’s interesting to note that NPC1 has received attention recently in many articles as a possible therapeutic target for antiviral techniques. NPC1’s role during viral infections has been studied pharmacologically using the hydrophobic amine U18666A and the antidepressant imipramine. The molecular pathophysiology of NPC illness is mimicked because both drugs block intracellular cholesterol transfer from lysosomes to the ER [24, 134]. To prevent cholesterol production and intracellular transport, U18666A is a cationic amphiphilic drug. U18666A suppresses cholesterol manufacture inside cells by decreasing levels of the enzyme oxidosqualene cyclase. It has been shown that U18666A blocks lysosomal cholesterol release by inhibiting NPC1, a cholesterol transporter. DENV, Ebola, hepatitis C, type 1 F-CoV, and SARS-CoV-1 replication have all been shown to be inhibited by the U18666A mutation [15, 21]. U18666A has a significant inhibitory effect on the S protein-driven entrance of SARS-CoV-1, MERS-CoV, and FCoV, all of which are coronaviruses [15, 135]. Blocking NPC1 is an efficient strategy for reducing SARS-CoV-2 infectiousness because it provides a multistep blockage of viral entrance via the PM or LE/L into host cells. Cepharanthine is an isoquinoline-containing cationic amphiphile with proven efficacy as an anti-inflammatory, anti-cancer, and antiviral treatment. Cepharanthine showed complete suppression of HIV and, most notably, the pangolin coronavirus, which has high sequence similarity to SARS-CoV-2, while additionally sharing the same host cell receptor (ACE2). It was discovered that cepharanthine had antiviral efficacy against SARS-CoV-2 in vitro [100, 136]. Scientists looked at the possibility that natural cepharanthine analogs might be effective anti-COVID-19 medications. The KNApSAcK database was mined for 24 compounds that resemble cepharanthine; molecular docking simulations were used to predict their binding affinities to target proteins like the SARS-CoV-2 spike protein, and main protease, NPC1, and the human two pore segment channel 2 (TPC2). A cell-based SARS-CoV-2 infection test was used to assess the selected analogs further. The effectiveness of cepharanthine and its derivative tetrandrine (TET) was also evaluated. By comparing their docking conformations, we may infer that the cepharanthine analogs have a common pharmacophore in their diphenyl ester moiety. Here, investigators present the results of an in silico docking investigation of natural cepharanthine analogs and an effort to test some of those analogs for their ability to inhibit SARS coronavirus type 2 replication in human cells. Although TET’s activity was lower than cepharanthine’s, it was nevertheless effective [137]. It is speculated that the chemicals cepharanthine and TET interact with the human lysosomal MPs NPC1 and TPC2, respectively. The molecular environments of target sites are hydrophobic because cholesterol and phosphatidylinositol 3, 5′-bisphosphate are the natural ligands of NPC1 and TPC2, respectively [45, 137, 138]. This attests to imipramine’s broad-spectrum antiviral action. The antiviral properties of imipramine were also preserved for other arboviruses, including the DENV, WNV, and ZIKV from the genus Flavivirus. These antiviral actions are anticipated to depend on the prevention of viral fusion with cell membranes and a potential direct impact on viral replication. The Flavivirus Class II viral fusion proteins and the Alphavirus E1 envelope glycoprotein, which facilitates viral fusion, have highly similar structural characteristics. As a result, DENV and WNV are susceptible to class II cationic amphiphilic medicines and need cellular cholesterol for effective fusion reactions. It is noteworthy that the need for cholesterol in the ZIKV life cycle has not yet been investigated. Still, researchers’ findings provide a first indication of how further to investigate this route [139, 140]. The use of a stable replicon cell line and time-of-addition studies showed that imipramine impeded the entrance and/or fusion of retroviral pseudo particles bearing the CHIKV envelope and hampered the post-fusion viral RNA replication stages [10] (Table 1).

## Conclusion

The host range and tissue tropism of viruses are primarily determined by viral entry receptors. Additionally, viruses employ host lipid metabolism and cellular lipids to enable their reproduction and dissemination. Due to their direct contact with EBOV GP, NPC1 are crucial for viral receptor activation, and this suggests that they may naturally affect the spectrum of filovirus hosts. Additional sequence variations in this gene that regulate susceptibility to filovirus infection and various virus types may be identified by broader surveys of NPC1 orthologs from vertebrates. Through genetic and biochemical characterizations, researchers found that NPC1 and NPC2 work together to transport cholesterol generated from LDL out of LE/LYSs. NPC1 is a lysosomal MP essential for cellular cholesterol homeostasis and susceptibility to infections caused by Ebola, other filoviruses, hepatitis virus, flaviviruses, SARS-CoV-2, SARS-CoV-1, and F-CoV. The current data shed light on the protein’s complex transport functions. Research into NPC1 in viral infection could lead to new broad-spectrum antiviral strategies for treating many enveloped virus infections by inhibiting cholesterol biosynthesis or uptake, stimulating cholesterol storage, and preventing cholesterol from being trafficked to the PM by modulating the function of cholesterol transfer proteins and suppressing, NPC1 results in systemic antiviral action, suggesting a novel treatment approach against SARS-CoV-2 and other emerging viruses. Therefore, further research is required before NPC1 may be used effectively as a therapeutic target for many types of viral infections.

## Data Availability

Not applicable.
